# Regulatory Mechanisms of Phenolic Acids in Metabolic Dysfunction-Associated Steatotic Liver Disease: A Review

**DOI:** 10.3390/antiox14070760

**Published:** 2025-06-20

**Authors:** Shengyu Zhang, Congcong Shen, Han Di, Yanhong Wang, Feng Guan

**Affiliations:** 1 School of Pharmacy, Heilongjiang University of Chinese Medicine, Harbin 150040, China; tt13892638691@163.com (S.Z.); scc16637647314@163.com (C.S.); 15846488827@163.com (H.D.); 2 Key Laboratory of Basic and Application Research of Beiyao, Ministry of Education, Heilongjiang University of Chinese Medicine, Harbin 150040, China

**Keywords:** phenolic acids, metabolic dysfunction-associated steatotic liver disease, non-alcoholic fatty liver disease, regulatory mechanism, hydroxycinnamic acids, hydroxybenzoic acids, antioxidant activity

## Abstract

Metabolic dysfunction-associated steatotic liver disease (MASLD), the leading chronic liver condition globally, constitutes a major etiological contributor to hepatocellular carcinoma (HCC). Its transition from steatosis to non-alcoholic steatohepatitis (NASH) involves progressive fibrosis, ultimately predisposing to HCC. The pathogenesis involves multifactorial interactions among genetic susceptibility, environmental triggers, and obesity-associated metabolic dysregulation. Crucially, the gut–liver axis serves as a pivotal regulatory mechanism in MASLD development. Current therapeutic strategies prioritize lifestyle interventions for metabolic syndrome management, while pharmacological options remain limited, underscoring the need for new therapies. Emerging evidence highlights phenolic acids—bioactive phytochemicals from medicinal plants—as multi-target agents against MASLD. These compounds demonstrate therapeutic efficacy via antioxidative modulation of stress, anti-inflammatory activity, and gut–liver axis regulation. This review synthesizes recent advances in natural phenolic acids for MASLD intervention, emphasizing their potential as preventive and therapeutic candidates. Their multimodal mechanisms may inform innovative drug development paradigms targeting MASLD pathogenesis.

## 1. Introduction

The liver, a vital and highly heterogeneous organ in the human body, serves as a central hub for diverse physiological processes, including nutrient and lipid metabolism, immune regulation, endocrine signaling pathway modulation, and drug catabolism [1]. Liver diseases are a leading cause of global morbidity and mortality. Among the key risk factors for hepatic metabolic disorders is a high-fat diet, which can contribute to the development of non-alcoholic fatty liver disease (NAFLD). Currently, NAFLD ranks among the most prevalent chronic hepatic disorders globally and is characterized by hepatic steatosis—excessive lipid accumulation exceeding 5% of hepatic mass without substantial alcohol intake [2]. Epidemiological studies reveal NAFLD prevalence in the global population is estimated to be 25.24% [3]. Since 2020, the international expert consensus has proposed replacing “NAFLD” with “Metabolic Dysfunction-Associated Fatty Liver Disease (MAFLD)” [4]. This renaming fundamentally stems from an in-depth understanding of the interaction mechanisms between fatty liver and metabolic disorders. The core motivation for the terminology shift lies in emphasizing the intrinsic link between the disease’s essence and metabolic dysfunction, while simultaneously abandoning the restrictive diagnostic framework of NAFLD that required the exclusion of alcohol consumption and other liver diseases. The new MAFLD definition focuses on the direct association between hepatic steatosis and metabolic abnormalities, with diagnostic criteria incorporating obesity, type 2 diabetes (T2DM), and metabolic dysregulations without mandatory exclusion of other liver diseases, thereby establishing a more pathologically grounded and standardized clinical management pathway [5]. In 2023, “A multisociety Delphi consensus statement on new fatty liver disease nomenclature” introduced the term “metabolic dysfunction-associated steatotic liver disease (MASLD)” and recommended discontinuing the use of “NAFLD” [6]. This updated terminology not only more accurately reflects the disease’s pathogenesis rooted in metabolic dysregulation but also highlights its systemic pathobiological impact. MASLD not only serves as a high-risk factor for hepatic events but also independently increases extrahepatic event risks—such as cardiovascular disease and cancer—by promoting atherogenic dyslipidemia and establishing systemic pro-inflammatory, pro-fibrotic, and pro-thrombotic microenvironment [7]. MASLD covers a wide range, typically including the benign mild steatosis of non-alcoholic fatty liver (NAFL). Patients with NAFL may develop necroinflammation characteristic of non-alcoholic steatohepatitis (NASH) accompanied by differing severities of inflammation, while more severe cases may progress with concomitant liver fibrosis [8]. Patients with liver fibrosis have the potential to develop cirrhosis and ultimately hepatocellular carcinoma (HCC) [9]. MASLD is recognized as the hepatic manifestation of metabolic syndrome. This condition is linked to metabolic abnormalities such as obesity, hypertension, hyperlipidemia, dyslipidemia, T2DM, and insulin resistance (IR) [10]. Furthermore, MASLD demonstrates significant correlations with endocrine disorders and cardiovascular disease-related complications such as polycystic ovarian syndrome, hypercortisolism, neuropathy, and other diseases [11].

**Figure 1:**
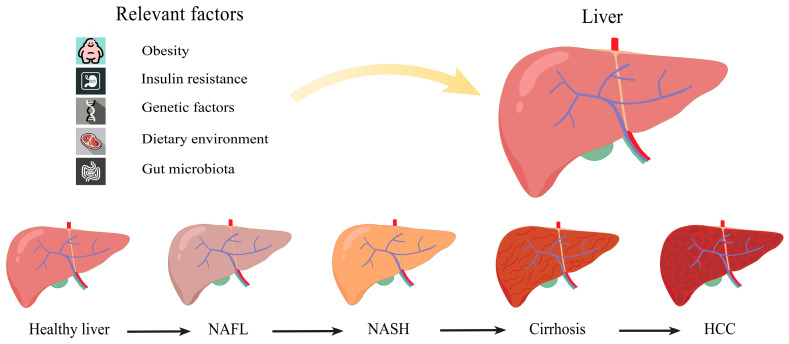
Contributing factors in MASLD pathogenesis.

Elucidating the pathogenesis of MASLD is essential for controlling MASLD and its comorbidities. The understanding of MASLD has evolved from being regarded as simple steatosis to the progressive establishment of the “two-hit” hypothesis [12]. In this model, hepatic steatosis represents merely the initial stage, where obesity and IR lead to lipid accumulation, constituting the “first hit” [13]. When excessive fat accumulates in the liver and the related protective mechanisms fail to completely eliminate fatty acids, the “second hit” occurs. During this phase, inflammation-induced cytokines and oxidative stress become critical factors, and uneliminated fatty acids cause cellular stress and activate inflammatory responses and fibrosis, ultimately leading to hepatocyte death [14]. With advancing research, the pathogenesis of MASLD has proven increasingly complex, rendering the “two-hit” hypothesis insufficient to explain the diverse metabolic alterations observed in MASLD [15]. Researchers also found that MASLD does not always correlate with metabolic syndrome. Beyond the inflammatory responses promoted by adipose tissue dysfunction due to IR and obesity, studies have revealed scenarios where inflammatory responses may precede steatosis onset. Notably, hepatic steatosis can also develop independently of IR [16]. The progression from simple steatosis to NASH may result from the failure of anti-lipotoxic protective mechanisms [17]. Multiple parallel processes—including gut microbiota alterations, genetic factors, and dietary environment—can collectively induce both hepatic steatosis and inflammation. This understanding has led to the emergence and widespread acceptance of the “multiple parallel hits” hypothesis as the prevailing pathogenic framework (Figure 1).

The progression of MASLD from NAFL to NASH and liver fibrosis, culminating in HCC, centers on intricate interactions among distinct hepatic cell types, including hepatocytes (HCs), Kupffer cells (KCs), hepatic stellate cells (HSCs), liver sinusoidal endothelial cells (LSECs), and their pathological alterations [18]. HC injury serves as the primary initiating factor, predominantly driven by lipotoxicity, oxidative stress, mitochondrial dysfunction, and related mechanisms. This process begins with excessive lipid accumulation and dysregulation of lipid metabolism pathways within HCs, thereby generating mediators of lipotoxicity that induce mitochondrial impairment, endoplasmic reticulum (ER) stress, and oxidative stress. These events directly trigger hepatocyte death and stimulate damaged HCs to release extracellular vesicles (EVs) carrying pro-inflammatory and pro-fibrotic mediators, ultimately promoting genomic instability [19]. Oxidative stress significantly exacerbates HC injury and death while suppressing proliferation, thereby compromising regenerative processes and perpetuating hepatic damage and inflammation. Lipopolysaccharide (LPS), intestinal flora, specific metabolites such as cholesterol, and damaged HCs activate liver-resident macrophages—KCs [16]. Reactive oxygen species (ROS) generation arises from two primary sources: (1) increased mitochondrial free fatty acid (FFA) in HCs; (2) activation of innate immune cells such as KCs [20]. Furthermore, KCs sustain the inflammatory milieu by releasing pro-inflammatory cytokines such as interleukin-6 (IL-6), interleukin-1β (IL-1β), factor-α (TNF-α). Concurrently, KCs secrete mediators including CCR5 ligands that directly regulate and activate HSCs’ proliferation and transdifferentiation [21]. HSCs serve as the primary producers of extracellular matrix (ECM) proteins and are recognized as central mediators in MASLD-related fibrogenesis. HSCs can be directly triggered by CCR5, and then synthesize and deposit collagen and other ECM components, driving liver fibrosis and cirrhosis [22]. LSECs—specialized fenestrated endothelial cells—play pivotal roles in maintaining normal hepatic homeostasis through metabolite exchange. During the transition from health to fibrosis, these cells exhibit progressive loss [23].

At present, the treatment methods for early MASLD patients are mainly to change their lifestyles, including dietary adjustments and physical exercise, aimed at achieving weight control and metabolic regulation [24]. However, such dietary and lifestyle interventions often prove unsustainable for widespread implementation across patient populations. Consequently, these patients require targeted pharmacological therapies specifically designed to ameliorate hepatic inflammation and fibrosis [25]. Currently, there remains no optimal pharmacological treatment for MASLD. Among them, while vitamin E demonstrates uncertain efficacy in preventing hepatic fibrosis and HCC, it remains a primary therapeutic option for nondiabetic patients. Conversely, diabetic NASH patients are typically managed with glucose-lowering agents approved for diabetes treatment [26]. Since the discovery of bidirectional gut–liver communication’s pivotal role in MASLD pathogenesis and progression, growing research has investigated the gut–liver axis, thereby opening new therapeutic possibilities for MASLD intervention [27]. At present, many drugs have also shown improvement effects on MASLD, such as metformin, statins, and the farnesoid X receptor (FXR) agonists (Table 1). Despite active and rapidly advancing drug development for MASLD, with numerous agents demonstrating potential, persistent challenges in safety and efficacy remain. Among them, silymarin and fucoxanthin are natural pharmaceutical extracts, and no obvious adverse events were found in clinical trials. This state has spurred growing research interest in herbal medicines and naturally derived chemical compounds [28]. An increasing number of studies have shown that Chinese herbs and their chemical extracts can be a new direction for the treatment of MASLD [29].

In the past few years, research and application of herbal medicines and their natural extracts have gained significant attention. Studies demonstrate that numerous natural products derived from plants can exert hepatoprotective effects through multiple pathways, with substantial evidence confirming the therapeutic potential of certain natural compounds against MASLD [39]. These phytochemicals offer distinct advantages including well-defined composition, simplified production processes, and enhanced controllability, positioning them as promising candidates for MASLD treatment strategies [40]. Consequently, investigating the therapeutic effects of natural extracts on MASLD has become a prominent research focus. Among various identified bioactive compounds, phenolic acids have demonstrated significant regulatory and ameliorative effects on MASLD [41]. Phenolic compounds are systematically categorized according to the quantity and structure of phenolic rings, primarily grouped as phenolic acids, lignans, stilbenes, flavonoids, and related compounds. Notably, phenolic acids constitute a subclass characterized by carboxyl-functionalized phenolic structures [42]. Phenolic acids are conventionally classified into two major categories: cinnamic acid derivatives and benzoic acid derivatives. These natural compounds predominantly exist as hydroxycinnamic acids (HCAs) and hydroxybenzoic acids (HBAs) [43].

The majority of phenolic acids exhibit remarkable bioactivity, primarily attributed to their potent antioxidant capacity [44]. Furthermore, these compounds also play an important role in MASLD by regulating intestinal microbiota composition, inhibiting inflammatory responses, and regulating lipid metabolism [45]. The pharmacologic activities of phenolic acids—particularly their antioxidative and anti-inflammatory characteristics—are intrinsically linked to their chemical structures. The structure of common phenolic acids is relatively similar, resulting in certain commonalities in their chemical properties and biological activities. However, variations in the number of substituent groups and their positions impart distinct biological activity profiles to individual compounds [46]. Phenolic acids exert antioxidant effects through multiple mechanisms. Research indicates their antioxidant activity correlates with both the density and three-dimensional configuration of hydroxyl moieties. The hydrophobic benzene rings and hydrogen-bonded phenolic hydroxyls enable these compounds to function as effective antioxidants [47]. The phenolic hydroxyl groups are excellent hydrogen donors, which can achieve oxidative stress mitigation by donating hydrogen atoms to free radicals and interfering with free radical generation, thereby reducing inflammation caused by oxidative stress [48]. Furthermore, phenolic compounds can chelate metal ions involved in free radical production, thereby contributing to their antioxidant activity and lipid metabolism regulation [49].

This comprehensive analysis synthesizes recent research advances regarding the therapeutic impacts of HCAs—including cinnamic acid (CiA), caffeic acid (CaA), ferulic acid (FA), p-coumaric acid (pCA), and chlorogenic acid (CGA)—along with HBAs such as gallic acid (GA), vanillic acid (VA), and protocatechuic acid (PCA) in MASLD. Furthermore, we clarify the pathways underlying the therapeutic actions of these phenolic acids against MASLD.

## 2. Regulatory Mechanisms of Phenolic Acids on MASLD

### 2.1. Hydroxycinnamic Acids

In the field of natural phenolic acids, HCAs represent the predominant subclass of phenolic compounds in terms of prevalence and structural diversity. According to structure, HCAs are structurally categorized into three primary variants: simple hydroxycinnamic acids, hydrogenated hydroxycinnamic acids, and polyhydroxycinnamic acids [50]. This section provides a concise overview of five predominant HCAs: CiA, pCA, CaA, FA, and CGA. Their respective regulatory mechanisms in MASLD pathogenesis are systematically summarized and critically analyzed (Table 2).

#### 2.1.1. Cinnamic Acid

CiA, systematically named 3-phenyl-2-propenoic acid, is predominantly found in *Cinnamomum cassia* (L.) D. Don and other botanical sources. Previous studies have extensively investigated CiA and revealed its diverse pharmacological properties. Studies have demonstrated that CiA exhibits multifaceted bioactivities, including anti-inflammatory, antioxidant, anti-tumor, anti-microbial, and antifungal activities [82]. Furthermore, CiA demonstrates multiple beneficial effects, including (1) enhancement of glucose tolerance and insulin secretion and (2) protective effects on multiple organs, particularly the spleen, heart, and liver.

Studies demonstrate that CiA ameliorates the infiltration of neutrophils and lymphocytes in hepatic tissues. The production of SCFAs increases with the specific microbiome regulated by CiA, and high-dose CiA can significantly increase the relative abundance and compositional ratios of *Firmicutes* [56]. SCFAs belong to pathogen-associated molecular patterns (PAMPs) provided by the intestinal microbiota. PAMPs are small molecular motifs in microorganisms, mainly containing LPS and lipoteichoic acid (LTA) [83]. They are detected through pattern recognition receptors (PRRs), which initiate innate immunological activation [84]. In patients with NASH, gut dysbiosis induces compromised gut barrier integrity, resulting in the excessive hepatic influx of PAMPs [85]. Upon entering the liver, PAMPs activate Toll-like receptors (TLRs) on macrophage surfaces, triggering massive secretion of inflammatory mediators and chemokines [86]. This inflammatory cascade exacerbates hepatocyte injury and promotes inflammatory cell infiltration, initiating and perpetuating inflammation, which serves as a pivotal driver in MASLD pathogenesis [87].

CiA demonstrates significant efficacy in regulating obesity and metabolic disorders. MASLD and IR exhibit reciprocal interactions: IR elevates blood glucose levels, stimulates lipogenesis, and aggravates hepatic fat accumulation. Simultaneously, IR induces the expression of tumor necrosis IL-6, IL-1β, TNF-α, and other pro-inflammatory cytokines, promoting hepatic inflammation and fibrosis. These interconnected mechanisms synergistically drive the development of MASLD [88]. CiA can reduce serum leptin concentration and pancreatic lipase activity, significantly lowering body weight in HFD-fed rats and treating hyperlipidemia [51]. Additionally, the accumulation of intrahepatic triglycerides (IHTGs) is one of the biomarkers of MASLD. CiA effectively inhibits lipogenesis and fatty acid intake while promoting hepatic fatty acid oxidation in vitro and in vivo, thereby reducing IHTG accumulation and ameliorating MASLD [53]. CiA enhances glucose uptake in IR adipocytes. Naturally extracted CiA and its derivatives are significantly effective in regulating IR in 3T3-L1 adipocytes [89]. Moreover, CiA can also promote the catabolism of 5-hydroxytryptamine (5-HT) and reduce its content in db/db mice [89]. 5-HT has been shown to induce the development of IR in both hepatocytes and adipocytes. In hepatocytes, the low-density lipoprotein receptor (LDLR) binds to apolipoproteins to maintain lipid clearance functions. CiA significantly upregulates LDLR expression and alleviates lipid metabolism disorders [54]. Transport proteins such as OATP1A2, NTCP, OCT1, MDR1, and MATE1 play crucial roles in hepatic metabolism. Studies on Linggui Zhugan Decoction have revealed a strong correlation between these transport proteins and CiA. Inhibition of these transport proteins leads to reduced CiA levels in the bloodstream [55].

As a multifunctional proinflammatory cytokine, TNF-α stimulates nuclear factor κB (NF-κB) signaling, subsequently upregulating the expression of downstream inflammatory mediators including IL-6 and IL-1β. This exacerbates hepatic inflammation and hepatocyte injury [90]. Elevated TNF-α levels in MASLD patients are strongly associated with disease initiation and advancement [91]. Trans-cinnamic acid (TCA), an isomer of CiA, significantly lowers body weight, adiposity, and lipid parameters in obesity animal models, while also lowering hepatic biomarkers and circulating concentrations of the inflammatory mediator TNF-α. Collectively, these findings demonstrate that TCA reduces inflammation and ameliorates MASLD by suppressing TNF-α and improving lipid metabolism [52].

**Figure 2:**
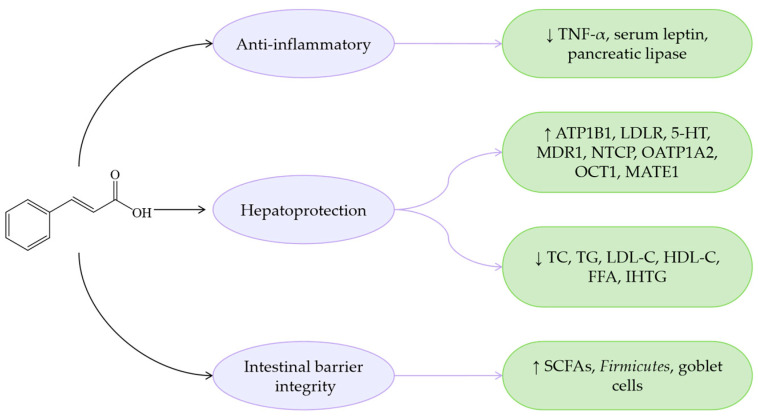
Mechanisms of CiA regarding MASLD. ↓ indicates a decrease in expression and ↑ indicates an increase in expression.

The antioxidant capacity of CiA is relatively modest, likely due to the absence of hydroxyl substitutions on its benzene ring. The mechanisms of CiA modulating MASLD are primarily attributed to its anti-inflammatory and metabolic regulation (Figure 2). For instance, CiA inhibits TNF-α expression, reduces the secretion of inflammatory mediators, improves IR, and decreases the accumulation of IHTG. Additionally, CiA regulates gut microbiota, thereby reducing the production of PAMPs, and exerts multi-faceted effects on MASLD progression.

#### 2.1.2. p-Coumaric Acid

Coumaric acid, systematically named 4-Hydroxycinnamic acid, naturally exists in three isomeric forms, among which pCA predominantly appears across botanical species with extensive dispersion, including fruits, vegetables, and herbal medicines [92]. pCA exhibits diverse multifaceted pharmacological attributes encompassing anti-tumor, antioxidant, and anti-inflammatory capacities. Moreover, pCA demonstrates high bioavailability and low toxicity [93]. pCA can protect against LPS-induced cellular damage by inhibiting oxidative stress and inflammatory responses [94]. pCA shows potential therapeutic efficacy in MASLD [57].

There is considerable evidence that pCA has the ability to improve MASLD. pCA demonstrates potent antioxidative properties and exerts hepatoprotective effects via modulation of the mitogen-activated protein kinase (MAPK) signaling axis and prevents damage caused by ROS [57]. Furthermore, pCA can ameliorate the negative effects of IR and up-regulate SIRT1. IR can increase the levels of total bilirubin, total cholesterol (TC) and triglyceride (TG), and concentrations of inflammatory mediators including TNF-α within hepatocytes in liver cells. The decrease of TG and TC is associated with the activation of hepatic lipase and the upregulation of lipid metabolic proteins. SIRT1 exerts anti-inflammatory actions through TNF-α suppression after deacetylation of NF-κB [58]. The combination of MET and PCA ameliorates MASLD by reducing liver lipid deposition such as TG, and inhibiting the NF-κB pathway to relieve inflammation [59]. The Si Miao Formula composition (berberine, betaine, caffeic acid, pCA, 2:2:1:1), which is rich in caffeic acid and pCA, can also effectively inhibit the development of MASLD [60]. pCA plays a positive role in the prevention and treatment of diseases associated with oxidative stress and lipid metabolism [95].

Peroxisome proliferator-activated receptors (PPARs) are ligand-activated transcription factors that orchestrate lipid homeostasis. PPARs consist of three subtypes: PPARα, PPARβ/δ, and PPARγ. PPARγ is critical for adipocyte formation and lipid accumulation, and it has garnered significant attention in the molecular mechanisms of MASLD and IR [96]. White adipose tissue serves as the main site for PPARγ agonists, thiazolidinediones (TZDs), which reduce blood lipid levels and elevate adipokine production, collectively enhancing insulin sensitivity. TZDs can reduce the risk of MASLD by 68% [97]. Research has found that pCA may function similarly to PPARγ agonists by regulating the induction of fatty acid catabolic enzyme expression, thereby attenuating hepatic lipid deposition [61].

**Figure 3:**
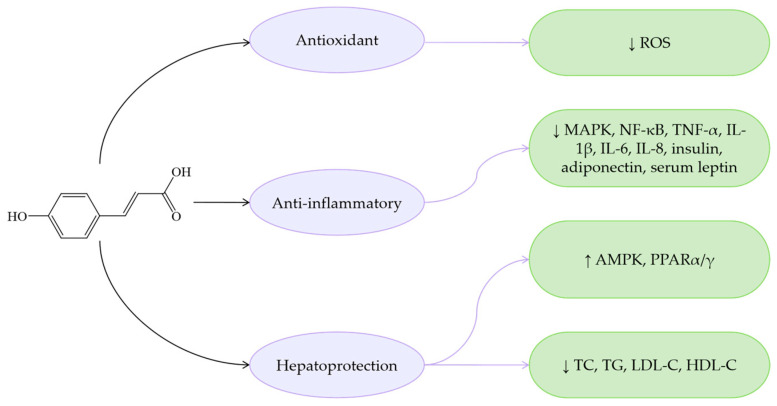
Mechanisms of pCA regarding MASLD. ↓ indicates a decrease in expression and ↑ indicates an increase in expression.

The antioxidant activity of hydroxycinnamic acids is related to the number of hydroxyl groups on the aromatic ring [98]. pCA demonstrates markedly enhanced antioxidant capacity compared to CiA, which has a positive effect on the prevention and treatment of MASLD (Figure 3). pCA lowers serum and hepatic levels of TG and TC, alleviates symptoms of IR, upregulates SIRT1 levels, and exerts PPARγ agonist-like effects in the liver. However, there are currently few studies on the mechanisms by which pCA regulates MASLD, and there is more focus on combination therapies involving various drugs. This has led to a lack of clarity regarding the exact mechanisms of pCA in MASLD.

#### 2.1.3. Caffeic Acid

CaA, systematically named 3,4-dihydroxycinnamic acid, exists in nature either as a free monomer or in complex oligomeric configurations bound to flavonoids and cell wall polymers. CaA is widely distributed in plants, including vegetables, fruits, and propolis [99]. CaA exhibits broad-spectrum bioactivities spanning antimicrobial, anti-inflammatory, and hepatoprotective effects. Its derivative, caffeic acid phenethyl ester (CAPE), is a primary constituent of propolis, which exerts significant therapeutic effects against diseases such as atherosclerosis and cancer by modulating inflammatory mediators including TNF-α, IL-1β, and IL-6 [100]. The antioxidant capacity, immunomodulatory activity, and anti-inflammatory activity of CaA can ameliorate inflammation and oxidative stress in MASLD.

CaA has a variety of biological activities. Studies on diabetic mice treated with CaA demonstrate its efficacy as a potent antioxidant. CaA inhibits low-density lipoprotein (LDL) lipid peroxidation, ameliorates atherosclerosis, significantly alleviates associated metabolic disorders, and blocks oxidative stress-induced hepatocyte damage in MASLD, thereby exerting hepatoprotective effects [64]. CaA reduces proinflammatory NF-κB signaling and adhesion molecule expression in vascular endothelial cells, while ameliorating obesity and associated metabolic dysregulation in rodent models. By inhibiting both PI3K/Akt signaling and MAPK pathways, CaA demonstrates anti-cancer efficacy and serves as a pivotal factor in mitigating hepatocellular damage and fibrotic progression linked to MASLD, and interferes with the transformation of MASLD into HCC [101]. CaA modulates metabolism via modulation of blood glucose levels and insulin sensitivity enhancement. These effects synergistically ameliorate IR and hepatic glucose and lipid metabolic dysregulation in MASLD [102].

In MASLD patients, the intestinal barrier cannot be overlooked, which is the biological boundary between the liver and gut microbiome [103]. Intestinal barrier integrity is critical for impeding the migration of pathogenic agents or toxic metabolites into the bloodstream [104]. In MASLD patients, increased intestinal permeability and gut dysbiosis allow PAMPs to translocate from the gut to the bloodstream. They enter hepatic circulation via the portal vein and exacerbate MASLD progression [105]. Ye et al. discovered that CaA alleviates DSS-induced intestinal inflammation by protecting intestinal barrier integrity and regulating gut microbiota [62]. This mechanism may indirectly influence MASLD progression. CaA suppresses T cells, macrophages, and neutrophils and inhibits NF-κB signaling to attenuate cytokine secretion and immune cell infiltration [63]. LPS, a PAMP known as bacterial endotoxin, is a surface component produced by Gram-negative bacteria [106]. It can cause metabolic endotoxemia and damage the integrity of the intestinal barrier [107]. Metabolic endotoxemia leads to a low-grade inflammatory state, which is one of the pathological features of MASLD [108]. CaA mitigates the elevated levels of LPS and several hepatic inflammatory markers. In addition, CaA reduces the prevalence of specific bacterial taxa enriched in HFD contexts. This suggests that CaA rebalances gut microbiota composition toward reduced LPS and prevents inflammatory responses in MASLD [109].

**Figure 4:**
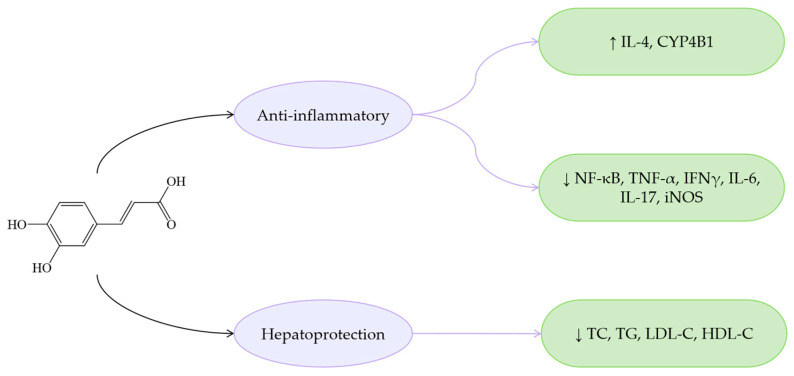
Mechanisms of CaA regarding MASLD. ↓ indicates a decrease in expression and ↑ indicates an increase in expression.

The location and number of hydroxyl groups in HCAs generally enhance their antioxidant capacity. Studies indicate that dihydroxy-substituted derivatives exhibit higher antioxidant activity. This phenomenon arises from ROS-mediated oxidation of HCA molecules, generating phenoxyl radicals, and phenoxyl radicals achieve stabilization through neighboring hydroxyl electron donors [110]. Additionally, CaA regulates the intestinal barrier, reduces the invasion of LPS, and regulates MASLD in many ways (Figure 4).

#### 2.1.4. Ferulic Acid

FA, systematically named 3-methoxy-4-hydroxycinnamic acid, is a phenolic acid compound widely distributed in the plant kingdom. It is commonly found in various fruits, vegetables, cereals, and cereal seeds [111]. FA exists in two isomeric forms, with the trans-isomer being the most prevalent. In recent decades, FA garnered substantial scientific interest owing to its robust bioactivities, particularly its antioxidant, anti-inflammatory, and anti-cancer abilities [112]. Recent studies have revealed that FA significantly improves MASLD through multi-target mechanisms. Due to its natural origin, favorable safety profile, and multi-target characteristics, FA represents a therapeutically promising agent targeting MASLD intervention.

Research has demonstrated that FA can inhibit MASLD progression through the gut–liver axis. Anatomically, hepatic–gut interaction occurs through the biliary tract. The molecular crosstalk between the liver and gut microbiome integrates signals as an interconnected system, collectively termed the gut–liver axis [113]. The gut microbiota metabolizes a variety of exogenous dietary components and endogenous substrates such as amino acids and BAs. The multiple metabolites produced in this process are transported to the liver through the portal vein, thereby affecting the liver [114]. In contrast, BAs and other hepatic products produced in the liver such as anti-microbial peptides can be transported to the gut through the bile ducts, where they have effects on gut microbiota, intestinal epithelial cells, and immune cells [115]. In summary, the direct and indirect interactions between the gut microbiota and the liver can trigger both local and distal responses to counteract and modulate the initiation and progression of diseases. FA has a protective effect against heat stress-induced intestinal epithelial barrier dysfunction. Specifically, FA enhances tight junctions (TJs) and microvilli structure, upregulating the expression of occludin, ZO-1, and E-cadherin, while reducing intestinal permeability [72]. Studies have shown that FA significantly reduces the TNF-α and IL-1β induced by APAP, inhibits the liver TLR4-mediated hepatic inflammatory responses, and effectively prevents APAP-induced liver injury [65]. TLRs, as PRRs of the innate immune system, play a pivotal role in microbial recognition and regulation of adaptive immune responses through their signal transduction pathways and immunomodulatory mechanisms. TLR4, a member of the TLR family, is activated by specific exogenous substances such as bacterial LPS and endotoxins, which trigger innate immune responses and inflammation [116]. TLR4 exacerbates the deterioration of MASLD by promoting lipogenesis, inflammation, and fibrosis [2]. Numerous studies have demonstrated the regulatory effect of FA on the TLR4 pathway. FA reduces inflammatory infiltration through the TLR4/NF-κB signaling pathway [68]. FA also can reduce LPS levels by inhibiting the TLR4 signaling pathway [117]. Furthermore, FA upregulates the abundance of *Dubosiella*, *Prevotella*, *Ruminococcus gnavus*, and *Faecalibaculum*, which can produce SCFAs and are critical for preserving gut permeability. Conversely, FA downregulates LPS-producing genera such as *Helicobacter* and *Mucor* [69]. FA preserves intestinal barrier function and ameliorates MASLD via gut microbiota-driven optimization of microbial metabolites [118].

Iron, a vital trace element, functions as a versatile catalytic cofactor in numerous enzymes involved in a variety of biological reactions and biosynthetic processes [119]. However, excessive iron triggers Fenton reaction-mediated ROS production, causing lipid peroxidation and cellular injury. Hepatic iron overload is a frequent comorbidity in MASLD [120]. FA exhibits iron-chelating capacity and a variety of physiological functions. FA improves HFD-induced metabolic syndrome owing to its antioxidative and anti-inflammatory capacities [66]. Through its chelating ability, FA activates the Keap1-nuclear factor-erythroid 2-related factor 2 (Nrf2) signaling pathway to suppress the generation of ROS and subsequently inhibit the expression of fatty acid synthase (FASN) [121]. FA activates the expression of CYP7A1, effectively preventing hypercholesterolemia while concomitantly inducing a secondary elevation of plasma BAs [70]. A recent study also showed that FA alleviates liver injury and reduces iron deposition in iron-overloaded mice. FA targets FASN to mitigate iron overload-induced lipid accumulation, while simultaneously chelating iron required for heme synthesis. This action lowers ROS levels, indirectly suppressing both the expression and activity of CYP7A1 protein, thereby regulating BAs’ metabolic disorders and ameliorating MASLD [67]. FASN is a central regulator of lipid metabolism that influences not only lipid metabolism but also glycolysis [122]. CYP7A1, a hepatic cytochrome P-450 enzyme, metabolizes various exogenous compounds including many drugs and natural products. As an iron-containing enzyme, CYP7A1 mainly regulates cholesterol homeostasis by converting cholesterol into BAs [123]. FA, as a natural iron chelator, provides novel therapeutic potential for MASLD. Comparative analysis reveals that in models demonstrating CYP7A1 upregulation, the drug dosage was doubled and treatment duration extended fivefold relative to models showing decreased CYP7A1 expression, which may partially explain these contradictory outcomes. Additionally, subtle differences exist in the experimental modeling approaches between studies. Given the limited available experimental data, the precise relationship between FA and CYP7A1 requires further investigation to reconcile these opposing conclusions.

PPARα, the first identified member of the PPAR family, serves as the master regulator of hepatic fatty acid oxidation and a key controller of lipid metabolism, and is one of the therapeutic targets of MASLD [124]. The synergistic and antagonistic relationship between PPARα and PPARγ offers novel insights into treating metabolic diseases. Studies demonstrate that FA significantly upregulates PPARα expression, reducing fat accumulation and liver weight in PPARα-deficient mice. FA enhances hepatic fatty acid β-oxidation and rate-limiting enzymes of ketone body biosynthesis to accelerate the decomposition of TG. Moreover, FA converts stored lipids into available energy substrates to increase energy expenditure [71], offering novel insights into managing metabolic diseases.

**Figure 5:**
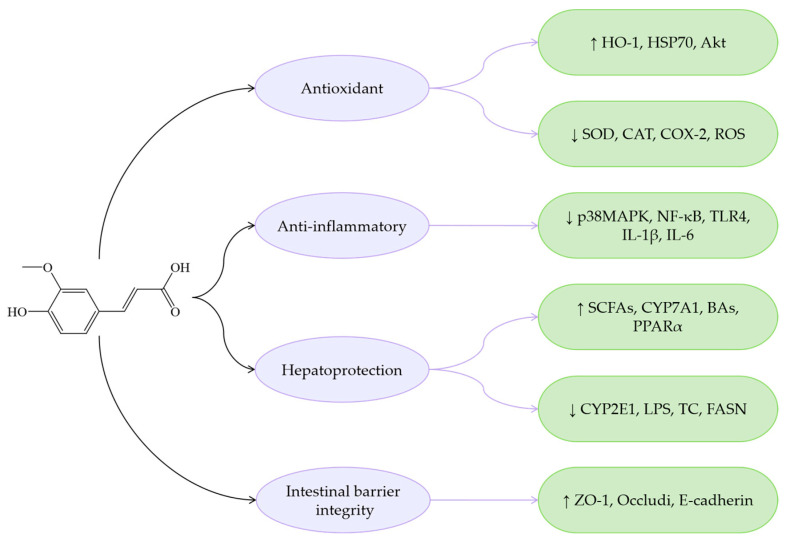
Mechanisms of FA regarding MASLD. ↓ indicates a decrease in expression and ↑ indicates an increase in expression.

Molecules with ortho-dihydroxyl or 4-hydroxy-3-methoxy groups (CaA and FA) have higher antioxidant activity than those lacking these structures [125]. This phenomenon may be attributed to the ability of such structures to stabilize free radicals more effectively and readily form five-membered-ring chelates with Fe^2+^ and Cu^2+^, inhibiting the Fenton reaction. Beyond mitigating MASLD progression through the gut–liver axis and alleviating oxidative stress and inflammation via iron chelation, FA also reduces NASH-induced elevations in TC, TG, and LDL levels while suppressing the NF-κB signaling pathway, ultimately ameliorating NASH-mediated liver injury (Figure 5) [126].

#### 2.1.5. Chlorogenic Acid

CGA is a functional phenolic acid abundantly present in plants such as honeysuckle and coffee beans [127]. It is a phenolic acid formed via esterification between CaA and quinic acid, belonging to the phenolic compounds synthesized through the shikimate pathway during plant aerobic respiration [128]. Research has revealed that CGA exhibits multiple diverse bioactivities, such as anti-inflammatory, anticancer, antimicrobial, antioxidant, and hypoglycemic effects, garnering significant attention in the biomedical community worldwide [129]. Moreover, CGA demonstrates remarkable hepatoprotective effects and can safeguard the liver against diseases such as MASLD through various mechanisms [130].

FXR functions as a BA-activated nuclear receptor in the liver and intestine. It plays a pivotal role in regulating cholesterol and BA homeostasis, including inhibiting BA synthesis and facilitating BA transport [131]. FXR controls multiple pathogenic pathways in MASLD. Beyond regulating BA synthesis, FXR modulates enterohepatic circulation, lipid and glucose metabolism, inflammation, and fibrosis [132]. Concurrently, FXR increases intestinal TJ expression and mucus production to maintain gut barrier integrity, prevent bacterial translocation, and preserve gut microbiota homeostasis [133]. Current research has found that the nuclear receptor FXR can reduce hepatic triglycerides to prevent MASLD onset, suggesting FXR agonists as a potential therapeutic strategy for MASLD treatment [134]. Studies demonstrate that CGA enhances the expression of FXR in the colon, modulates BA metabolism, and enriches BAs in the serum of mice [80]. Other studies indicate that the combination of Geniposide and CGA activates FXR signaling and increases the expression of FXR, BSEP, and SHP in liver tissues of NASH mice, as well as enhancing FGF 15 expression in ileal tissues [81].

Glucagon-like peptide-1 (GLP-1), an incretin hormone, is produced by intestinal enteroendocrine L-cells and is rapidly degraded primarily by dipeptidyl peptidase-4 secreted from the liver [135]. GLP-1 not only regulates glucose metabolism but also modulates lipid metabolism, reduces inflammation, and inhibits apoptosis. Experiments have shown that GLP-1 secretion is significantly decreased in MASLD patients. Notably, IR, hyperinsulinemia, and other related diseases are present in nearly all patients, and these metabolic disturbances are more severe in NASH compared to MASLD [136]. The gut microbiota is closely linked to incretin effects, and its alterations can influence the differentiation and apoptosis of intestinal epithelial cells, thereby modulating GLP-1 secretion [137]. In addition, SCFAs, as metabolites of gut microbiota, play a regulatory role in GLP-1 secretion [138]. In HFD-induced MASLD mice, CGA treatment altered gut microbial diversity, specifically reducing the abundance of *Blautia*, *Akkermansia*, and *Sutterella* genera while increasing SCFA-producing bacteria, enhancing butyrate and other SCFA production [75]. This phenomenon leads to increased GLP-1 levels. Rotenone-induced PD mouse models suggest that CGA promotes GLP-1 secretion [139]. Relevant clinical studies have also shown that CGA elevates GLP-1 production by enriching beneficial gut bacteria and enhancing SCFA generation, thereby ameliorating hepatic steatosis and inflammation [140].

LPS initiates innate immune responses. When LPS is uncontrolled, it may lead to severe diseases, including MASLD [141]. Following intestinal barrier disruption, LPS translocates and activates TLR4 to induce inflammation, upregulating TLR4 expression on immune cells, hepatocytes, and adipocytes [142]. This phenomenon may elevate circulating LPS levels, defined as metabolic endotoxemia—a condition associated with metabolic disturbances including dyslipidemia, IR, and MASLD [143]. TLR4 triggers signaling cascades involving IκB phosphorylation and NF-κB-mediated inflammatory responses, which promotes hepatic fibrosis and drives MASLD progression to NASH and HCC [144]. Studies demonstrate that CGA significantly reduces the production of TNF-α, IL-1β, and IL-6, while suppressing TLR4 expression and LPS-induced phosphorylation of NF-κB and IκB [76]. CGA protects intestinal integrity and alleviates inflammatory responses by inhibiting the growth of *Bacteroides* and their LPS production [77]. Moreover, CGA directly binds to myeloid differentiation primary response 88 (MyD88) to competitively block TLR4/MyD88 interaction, thereby reducing LPS-TLR4-MyD88-mediated hepatic inflammation during NASH progression [73].

Disruption of the intestinal barrier exacerbates the progression of metabolic disorders (such as IR) and allows pathogenic bacteria and endotoxins to enter the hepatic circulation. Upon reaching the liver, these pathogens engage receptor-specific binding to initiate signaling cascades such as NF-κB, triggering the release of reactive oxygen species and pro-inflammatory cytokines. This cascade leads to hepatitis, hepatic fibrosis, and steatosis, each contributing substantially to MASLD pathogenesis [145]. CGA enhances intestinal barrier function through multiple mechanisms. Firstly, CGA directly suppresses mucosal inflammation and downregulates pro-inflammatory mediators. CGA inhibits the TLR4 and NF-κB pathways, activates the Nrf2/heme oxygenase-1 (HO-1) signaling pathway, and downregulates key inflammatory molecules in the jejunal and ileal mucosa [74]. Additionally, CGA mitigates LPS-induced intestinal barrier damage and gut inflammation [146]. Secondly, CGA enhances antioxidant capacity. Research demonstrates that CGA suppresses NF-κB signaling by inhibiting p38MAPK, thereby reducing chronic stress-induced intestinal damage [78]. Furthermore, CGA mitigates oxidative damage, inflammatory responses, and apoptosis by activating Nrf2/HO-1 signaling while suppressing the MAPK/NF-κB/NLRP3 axis [147]. Thirdly, experimental studies reveal that CGA increases the villi height of duodenal, jejunal, and ileal and the villus crypt depth ratio in the jejunum and ileal mucosa of weaned piglets. CGA upregulates the expression of the TJ protein [148]. Separate research confirms that CGA enhances the expression of both TJ protein ZO-1 and occludin in intestinal cells and inhibits the permeability of the inflammatory intestinal tract [79]. CGA can also protect the intestinal barrier by suppressing the Rho-associated kinase 1 (ROCK1) and myosin light chain kinase (MLCK) signaling pathway and alleviating ER stress, which collectively upregulate TJ protein expression [149].

**Figure 6:**
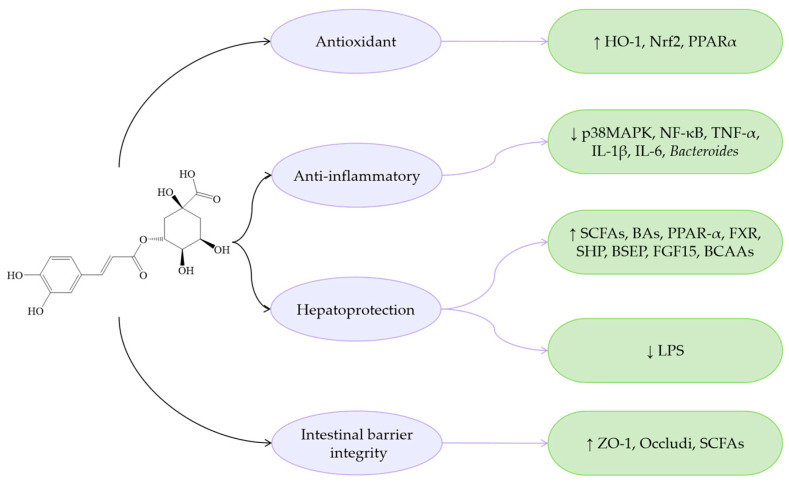
Mechanisms of CGA regarding MASLD. ↓ indicates a decrease in expression and ↑ indicates an increase in expression.

In summary, CGA has abundant mechanisms to improve MASLD (Figure 6). In addition to its antioxidant effects, CGA modulates inflammation and lipid metabolic pathways via the gut–liver axis while relieving the permeability of the intestinal barrier. Specifically, CGA inhibits the TLR4/NF-κB pathway to downregulate pro-inflammatory cytokines. CGA enhances FXR expression to improve BA metabolism in both the gut and serum, increases the abundance of beneficial bacteria to promote SCFA production and elevate GLP-1 levels, and alleviates ER stress. These findings demonstrate that CGA represents a promising therapeutic candidate for MASLD prevention and treatment.

### 2.2. Hydroxybenzoic Acids

HBAs are categorized into four major groups depending on their hydroxybenzoic acid chemical structures: simple hydroxybenzoic acids, polyhydroxybenzoic acids, hydroxybenzoate salts, and hydroxybenzoic acid glycosides [50]. Three common HBAs are selected in this section: PCA, VA, and GA (Table 3).

#### 2.2.1. Protocatechuic Acid

PCA, systematically named 3,4-dihydroxybenzoic acid, is widely distributed in vegetables, fruits, and medicinal herbs, with particularly high concentrations found in *Salvia miltiorrhiza* Bunge [168]. PCA has been proven to possess multiple pharmacological activities, including antioxidant, anti-inflammatory, anti-tumor, and hepatoprotective effects [169]. However, the regulatory mechanisms of PCA in MASLD are still not well elucidated.

MASLD is closely associated with inflammation and excessive oxidative stress. Given its strong antioxidative and anti-inflammatory effects, PCA effectively upregulates Nrf2 signaling to enhance antioxidant enzymes and phase II enzymes, effectively preventing menadione-induced oxidative stress in rats [150]. PCA inhibits NF-κB activation and reduces the levels of TNF-α and IL-6 to improve hepatic steatosis [153]. Furthermore, PCA protects against H_2_O_2_-induced hepatic oxidative damage in mice by boosting Nrf2 expression, shielding hepatocytes from ROS-mediated apoptosis under oxidative stress conditions [151].

PCA ameliorates MASLD by alleviating IR. With its strong affinity for acyl-CoA synthetase family member 3 (ACSF3), PCA activates sirtuin 3 (SIRT3) by inhibiting ACSF3-mediated fatty acid metabolism, thereby preventing MASLD development [152]. As a key regulator of fatty acid metabolism during MASLD, SIRT3 prevents metabolic dysfunction by suppressing ACSF3-induced disorders in fatty acid metabolism [170]. Red rice bran extract, rich in PCA, alleviates MASLD and dyslipidemia through the regulation of lipid-regulating gene expression [154]. PCA may mitigate MASLD by enhancing insulin sensitivity. Specifically, PCA inhibits *Enterococcus faecalis* and upregulates the expression of FGF1, insulin receptor substrate 1 (IRS1), insulin receptor substrate 2 (IRS2), and insulin-like growth factor binding protein-2 (IGFBP-2) to potentiate insulin signaling pathways [155]. At the same time, PCA regulates serum cholesterol levels, high-density lipoprotein (HDL) levels, and thiobarbituric acid reactive substances (TBARS) levels to prevent hepatic lipid accumulation. Notably, most of the downregulated lipid metabolites by PCA are known to induce or participate in IR and promote MASLD progression [156].

**Figure 7:**
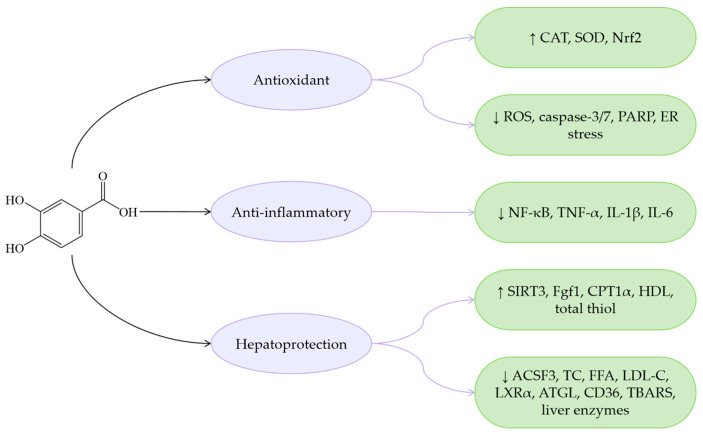
Mechanisms of PCA regarding MASLD. ↓ indicates a decrease in expression and ↑ indicates an increase in expression.

Overall, PCA exhibits similar bioactive properties to other phenolic acids in terms of anti-inflammatory and antioxidant effects, while demonstrating distinct advantages in lipid metabolism regulation (Figure 7). Specifically, PCA reduces *Enterococcus* abundance, thereby alleviating *Enterococcus*-induced fatty liver, IR, and IL-1β production. Furthermore, PCA improves MASLD by enhancing insulin sensitivity. Due to strong hydrogen bonding and van der Waals interactions, PCA shows high binding affinity for SIRT3. Compared to benzoic acid, protocatechinic acid with two additional hydrophilic groups provides more interaction sites with SIRT3 [152], enabling effective modulation of the SIRT3/ACSF3 pathway to ameliorate MASLD.

#### 2.2.2. Vanillic Acid

VA, chemically designated as 4-hydroxy-3-methoxybenzoic acid, is a phenolic compound abundantly present in vanilla beans and *Angelica* species. Owing to its aromatic properties, VA is commonly utilized as a flavoring agent and fragrance ingredient [171]. It has been demonstrated that VA exhibits multiple pharmacological activities, including antioxidant, anti-inflammatory, and hepatoprotective effects [172]. VA can inhibit inflammatory cytokines, prevent hepatic fibrosis, and activate the AMPK pathway, which can affect almost all metabolic abnormalities associated with MASLD [173].

VA upregulates the insulin signaling and lipid metabolic pathway-associated protein levels in HFD-fed rats, ameliorating insulin resistance through enhanced hepatic insulin signaling and modulated inflammatory pathways [159]. VA affects the NF-κB signaling pathway to reduce inflammation, increasing extracellular matrix component expression while downregulating pro-inflammatory cytokines such as TNF-α, IFN-γ, and IL-1β, demonstrating significant anti-inflammatory efficacy [158]. VA boosts antioxidant enzyme activity, such as SOD and CAT, which scavenge ROS and mitigate cellular lipid peroxidation [157]. Additionally, VA exerts hepatoprotective effects by upregulating hepatic FGF21 expression [160]. Liver fibrosis is the main pathological progression from MASLD to HCC. In MASLD-associated hepatitis models, MIF and extracellular receptor CD74 signaling pathways show pro-fibrotic effects. VA suppresses liver fibrosis by suppressing autophagy in hepatic stellate cells through inhibition of the MIF/CD74 axis [161].

VA plays a protective role in maintaining intestinal barrier integrity. Studies demonstrate that VA increases the *Firmicutes/Bacteroidetes* ratio and reduces the relative abundance of certain *Prevotellaceae* species, while upregulating beneficial microbiota such as *Lachnospiraceae* and *Muribaculum*. These modifications improve the gut microenvironment, decrease LPS production, and enhance occludin expression [162]. Ferroptosis refers to iron-mediated cell death triggered by lipid peroxidation [174]. VA exerts inhibitory effects on ferroptosis by binding to carbonic anhydrase IX (CA9), which disrupts the interaction between insulin-induced gene 2 (INSIG2) and SREBP cleavage-activating protein (SCAP). This disruption triggers SCAP-SREBP1 complexes migration from the ER to the Golgi apparatus. The activated SREBP1 significantly enhances the transcription of stearoyl-CoA desaturase-1 (SCD1), thereby inhibiting ferroptosis and preventing excessive intestinal epithelial cell death [163].

**Figure 8:**
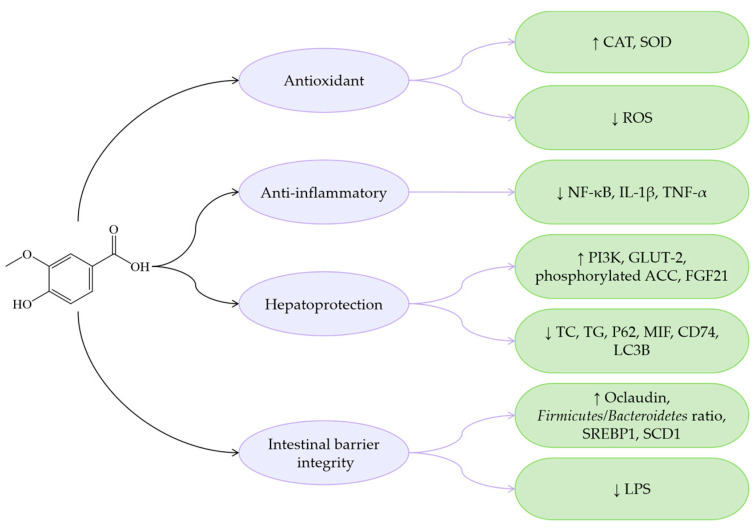
Mechanisms of VA regarding MASLD. ↓ indicates a decrease in expression and ↑ indicates an increase in expression.

VA alleviates MASLD by suppressing the NF-κB-mediated inflammatory cascade and lowers pro-inflammatory mediators such as IL-2, IL-6, and TNF-α, while also scavenging ROS, activating the AMPK pathway, and decreasing lipid accumulation to improve IR-related metabolic disorders. Furthermore, VA ameliorates liver fibrosis via the MIF/CD74 pathway stimulation. VA modulates gut microbiota composition and inhibits CA9-mediated ferroptosis to maintain intestinal barrier integrity, thereby preventing unresolved inflammatory progression (Figure 8).

#### 2.2.3. Gallic Acid

GA, chemically known as 3,4,5-trihydroxybenzoic acid, is predominantly found in various dietary sources including tea, with concentrations ranging from 0.1% to 2% across different tea varieties [175]. Studies report that GA possesses diverse bioactivities, such as antioxidant and anti-inflammatory actions, while it demonstrates therapeutic potential against metabolic disorders such as MASLD [176].

GA reduces levels of various metabolites such as fatty acids, improves glycolysis, and protects the liver by decreasing the excretion of taurine and glycine, while also enhancing choline metabolism and amino acid metabolism. Through these multifaceted mechanisms, GA comprehensively ameliorates the metabolic disturbances induced by HFD in MASLD mice [177]. GA critically contributes to inhibiting the progression of MASLD to HCC. It downregulates mRNA expression of sterol regulatory element-binding protein-1c (SREBP-1c) and liver X receptor alpha (LXRα) while enhancing phosphorylation of AMP-activated protein kinase (AMPK), thereby ameliorating hepatic lipid accumulation [165]. As a central regulator of cellular metabolism homeostasis, AMPK phosphorylates and inactivates acetyl-CoA carboxylase (ACC), inhibits hepatic lipid accumulation, and promotes mitochondrial fatty acid oxidation [178]. At the same time, AMPK activates PPARα to enhance fatty acid uptake. AMPK serves as a pivotal factor in metabolic disorders including MASLD; AMPK activation is now considered a viable approach for managing metabolic diseases [179]. Through activating the AMPK-ACC-PPARα pathway, GA decreases hepatic lipid deposition and mitigates lipotoxic effects both in vitro and in vivo. Additionally, GA mitigates excessive ROS production in the liver by comprehensively improving mitochondrial function [166]. In addition, epidermal growth factor receptor (EGFR) stimulation has been identified as a therapeutic target for dyslipidemia and MASLD [180]. Research demonstrates that GA specifically targets the extracellular domain of EGFR to activate the EGFR-extracellular signal-regulated protein kinase (EGFR-ERK1/2) signaling pathway. Concurrently, studies reveal that low-density lipoprotein cholesterol (LDL-C) accumulation is associated with activation of the EGFR-ERK1/2 pathway [167]. Epidemiological studies have shown that the prevalence of MASLD is related to elevated LDLR protein levels [181]. Consequently, GA can enhance LDL-C uptake and ameliorate MASLD by prolonging LDLR mRNA half-life, increasing its stability, and promoting LDLR accumulation.

Regarding anti-inflammatory effects, GA potently inhibits the NF-κB signaling pathway and upregulates IL-4 and IL-10 expression, while downregulating pro-inflammatory cytokines including TNF-α, IL-1, IL-6, and IL-12 [164]. GA attenuates inflammation via regulation of the TLR4/MyD88/TRIF signaling pathway [182]. Additionally, through enhancing hepatic antioxidant capacity and suppressing ROS-NF-κB-TNF-α inflammatory cascades, GA demonstrates therapeutic efficacy in MASLD rat models [183].

**Figure 9:**
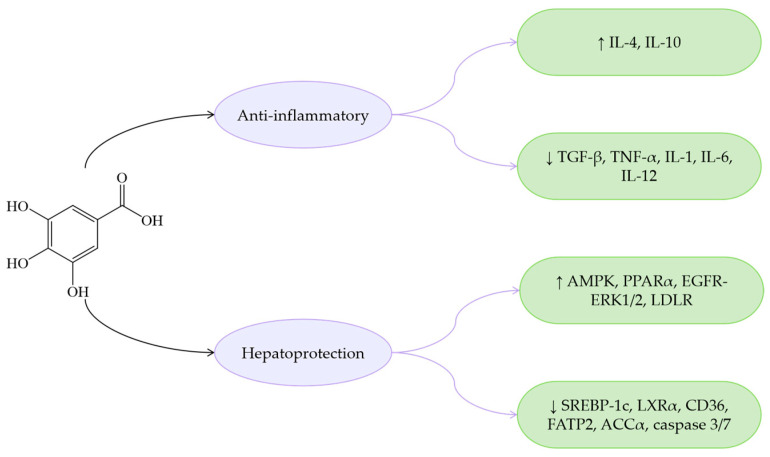
Mechanisms of GA regarding MASLD. ↓ indicates a decrease in expression and ↑ indicates an increase in expression.

These findings collectively demonstrate that GA effectively ameliorates MASLD through multiple mechanisms: activating the AMPK-ACC-PPARα signaling axis to enhance LDLR mRNA stability and reduce hepatic lipid accumulation, regulating lipid metabolism, exerting antioxidant effects, suppressing ROS, and attenuating inflammation via the NF-κB pathway inhibition (Figure 9). GA, as a natural dietary compound with MASLD-improving potential, offers promising research directions for MASLD treatment.

## 3. Bioavailability of Phenolic Acids

The absorption efficiency of phenolic acids is determined by their chemical structures, such as the ester group in CGA which significantly impairs its absorption. After ingestion, only a portion of phenolic acids is absorbed in the upper gastrointestinal tract, with up to two-thirds of the ingested dose reaching the colon for microbial decomposition by the gut microbiota [47]. Distinct absorption mechanisms exist among different phenolic acids: pCA and FA undergo efficient absorption in Caco-2 cells primarily via monocarboxylate transporter (MCT)-mediated active transport. Structurally analogous compounds, such as FA and CiA, compete for the same transporters. Bioavailability comparisons reveal that pCA exhibits superior absorption to FA, with its free form rapidly absorbed across the stomach, jejunum, ileum, and colon [184]. Additionally, pCA demonstrates significantly higher absorption efficiency than GA in rats, reflecting their divergent absorption properties attributable to MCT selectivity toward specific phenolic acids [185]. GA, CaA, and CGA show low affinity for MCT. These phenolic acids typically rely on paracellular diffusion for more efficient passive absorption [186]. Consequently, pCA and FA achieve higher absorption efficiencies than these compounds.

The distribution of phenolic acids within the organism and their high absorption bioavailability are crucial for conferring health benefits. Following absorption, only a minor fraction of CaA reaches the systemic circulation [187]. FA is distributed across all major organs, with particularly high accumulation in the kidneys [188]. pCA is substantially absorbed via passive diffusion in the duodenum and subsequently distributes to vital organs including the brain, heart, liver, and kidneys [189]. GA is initially absorbed through the gastrointestinal tract and primarily localizes in renal tissues [182]. The distribution kinetics of these phenolic acids and their metabolites indicate predominant renal accumulation. While first-pass metabolism significantly influences bioavailability and consequently bioefficacy, the limited gastrointestinal absorption and low hepatic concentrations of phenolic acids suggest a restricted first-pass effect, enabling extensive post-absorption distribution to peripheral organs [190].

The absorption and distribution of HCAs are metabolism-dependent. Following ingestion and absorption, phenolic acids undergo conjugation via glucuronidation, methylation, and sulfation reactions; for instance, GA exists in vivo as diverse glucogallin derivatives [182]. These metabolic modifications (glucuronidation, sulfation, methylation, hydrogenation) occur in both intestinal epithelial cells and the liver. Furthermore, intestinal metabolism involves gut microbiota that biotransform ingested phenolic acids into metabolites, often exhibiting enhanced bioactivity and superior absorption compared to parent compounds [191]. Studies demonstrate that despite the extensive metabolism of FA and CaA, their metabolites retain potent biological activities [192]. FA primarily exists in the glucuronidated form at the serosal side, with glucuronides and sulfoglucuronides constituting the most abundant FA metabolites in plasma [193]. Post-absorption, these phenolic acids circulate predominantly as conjugated forms in human plasma. VA, a methylated metabolite of PCA, is present in plasma either in free form or conjugated through glucuronidation/sulfation [194]. Similarly, CaA and CGA circulate as glucuronidated/sulfated derivatives, while m-coumaric acid and 3-(3-hydroxyphenyl) propionic acid emerge as primary colonic microbial metabolites of CaA and CGA [195].

Phenolic acids undergo rapid and extensive metabolism, leading to a swift decline in plasma concentrations, with VA returning to baseline within 4 h post-ingestion [196]. In rats, urinary excretion of FA occurs rapidly, reaching a plateau 1.5 h post-administration, whereas in humans the plateau occurs between 7 and 9 h after consumption [193]. PCA appears to exhibit a relatively longer half-life [189]. Excretion primarily proceeds via biliary and urinary routes. Biliary excretion shows significant species divergence due to gallbladder presence in humans versus rats. Macromolecular-conjugated metabolites are preferentially secreted into bile, where intestinal β-glucuronidase hydrolyzes them into free aglycones for potential reabsorption, although this pathway contributes minimally [197]. Urinary excretion serves as the dominant route, with low-molecular-weight conjugates directly eliminated renally. Excretion proportions vary by phenolic acid type. Ingested HCAs, except CGA, are predominantly excreted in urine as free, glucuronidated, and sulfated forms within 0–6 h. Total 48-h urinary recovery rates rank as follows: FA > CaA > pCA >> CGA [198].

Studies indicate that CaA exhibits low oral bioavailability in rats, poor intestinal absorption, and limited permeability in Caco-2 cells [199]. Different phenolic acids exhibit distinct absorption mechanisms due to structural variations. Post-absorption distribution demonstrates tissue-specific selectivity, FA, CaA, and their metabolites predominantly accumulate in kidneys, whereas pCA rapidly distributes to the brain, heart, liver, and kidneys. CGA metabolites such as m-coumaric acid achieve widespread distribution owing to limited first-pass metabolism [47]. Collectively, PCA and FA demonstrate superior bioavailability owing to efficient active transport, extensive tissue distribution, and bioactive metabolites, whereas CaA, despite its poor absorption, generates metabolites with significant biological activity.

## 4. Safety Evaluation of Phenolic Acids

The potential toxicity of a substance refers to its capacity to induce physiological imbalance upon exposure. Notably, no chemical compound is entirely free of toxicity, making toxicological studies essential for all bioactive molecules [200]. Phenolic acids are known for their antioxidant, anti-inflammatory, and anti-microbial properties. Although phenolic acids have been proven to have a natural advantage of low toxicity, phenolic acids still possess inherent toxic potential, necessitating rigorous therapeutic safety evaluations before clinical application [201].

CiA is a low-toxicity compound, which exhibits lower toxicity than cinnamaldehyde and cinnamyl alcohol. In vivo studies further demonstrate its safety profile: long-term administration in rats showed no hepatic function alterations, suggesting no cumulative toxicity. Even when the dose exceeds 300 times the daily human intake, CiA is not embryonically toxic to rats [202]. The CYP26 hepatotoxicity score of CiA is 0, which indicates its low toxicity. ADMET and DFT analyses confirm CiA’s negligible toxicity and reactivity [203]. pCA exhibits low toxicity in mice, with a median lethal dose (LD_50_) of 2850 mg/kg [204]. pCA demonstrates reparative capacity for liver injury at 20 mg/kg, with an optimal dosage of 100 mg/kg. The 28-fold dose difference indicates the favorable safety of pCA. Early experiments demonstrated that CaA had an LD_50_ of 4850 mg/kg, exceeding clinical dosages by more than 50-fold [205]. In reproductive and developmental mice toxicity studies of CaA, high doses (150 mg/kg/day) caused increased fetal weight but showed no maternal toxicity, fetal malformations, or postnatal developmental effects in offspring [206]. The oral toxicity of FA is comparable to that of pCA. The LD_50_ values for FA in rats are 2445 mg/kg (male rats) and 2113 mg/kg (female rats) [193]. Studies reveal cell-type-dependent toxicity that FA shows no significant toxicity toward platelets, leukocytes, or erythrocytes, but exhibits marked inhibitory effects on the proliferation of human monocytes and colon cells [207]. This may relate to their bioavailability: both FA and pCA exhibit higher bioavailability than CaA, resulting in lower LD_50_ values than CaA. Additionally, FA can cause damage to the kidney [208], whereas pCA has demonstrated renal protective effects. CGA is also a low-toxicity phenolic acid compound. Evaluations through cellular and animal models confirm its safety profile, demonstrating no adverse effects at in vitro concentrations not exceeding 400 μg/mL and oral doses as high as 15,000 mg/kg in vivo [209]. In a study of safety pharmacological effects, CGA has been studied by assessing cardiac toxicity, blood–brain barrier permeability, and gastrointestinal function, consistently supporting its safety [210]. Additionally, CGA effectively alleviates triptolide-induced acute liver injury with a mean median effective dose (ED_50_) of 50 mg/kg [136]. Regarding bioavailability, CGA possesses the lowest bioavailability among phenolic acids due to its ester group and weak affinity for MCT. This may partially explain CGA’s enhanced safety profile. Combined with CGA’s ED_50_, its therapeutic index (TI) of 300 highlights the potent bioactivity and safety of CGA.

Acute toxicity studies in mice indicate that PCA is safe, although no mortality was observed at these levels [168]. PCA exhibits dose-dependent dual pharmacological effects. PCA plays a therapeutic role at low doses and promotes toxicity at high doses. The LD_50_ in mice is 3500 mg/kg via intravenous injection, with an oral toxic dose of 500 mg/kg; post-intoxication, no animal mortality was observed [211]. In subacute toxicity studies of VA, high-dose administration (1000 mg/kg/day) caused no adverse effects in rats, with no observed toxicity to leukopoiesis, erythropoiesis, or visceral organs [212]. Therefore, clarifying PCA’s metabolic stability and primary phase metabolism pathways is crucial for understanding its therapeutic window and safe clinical dosing. The LD_50_ for VA in rats reached 5020 mg/kg and 2691 mg/kg in mice via intraperitoneal injection [213]. Pharmacokinetic data on VA, particularly its oral absorption kinetics, remain limited, posing constraints for safety assessments. In cellular experiments, GA exhibits mild cytotoxicity toward specific cell lines such as Caco-2, L929, and U937 at concentrations exceeding 200 μM. GA exhibits safety and efficacy across most cell types at low doses [214]. In vivo studies confirm GA’s low toxicity profile. Acute toxicity tests in albino mice revealed that GA’s LD_50_ exceeded 2000 mg/kg, and subacute toxicity assessments at 900 mg/kg showed no alterations in behavioral, morphological, or histopathological parameters [182]. GA shows relatively good oral absorption but undergoes rapid metabolism, leading to a short exposure time of the parent compound. This rapid metabolic inactivation may partially explain its observed low systemic toxicity, though it may also limit sustained efficacy.

It is evident that the aforementioned phenolic acids generally exhibit favorable safety profiles. These compounds are characterized by high bioavailability (FA, pCA) and broad therapeutic windows (LD_50_ > 2000 mg/kg), and demonstrate robust safety foundations. However, current safety evaluation systems remain limited, with existing data primarily derived from rodent models and in vitro studies, while long-term human toxicity, reproductive toxicity, and interspecies metabolic variations are inadequately characterized. Diverse MASLD research models and inconsistent dosing regimens across studies have hindered systematic comparisons of relative pharmacodynamic potency and therapeutic indices among phenolic acids, necessitating further experimental validation. Nevertheless, existing evidence allows preliminary screening of promising phenolic acid candidates for clinical translation. Distinct safety variations exist among phenolic acids. Despite FA’s high bioavailability, it carries defined nephrotoxicity risks, warranting cautious use in chronic nephritis or renal impairment patients. Although renowned for antioxidant activity, phenolic acids may act as pro-oxidants [215]. PCA possesses a narrow therapeutic window, with high doses inducing oxidative toxicity. CGA enhances hypoglycemic effects by promoting GLP-1 secretion but consequently carries hypoglycemia risk. Additionally, the estrogenic activity of phenolic acids may promote breast cancer development [216]. At low doses, GA, CaA, and FA stimulate Nrf-2 and suppress ROS generation, yet paradoxically exhibit carcinogenic potential [217]. Nevertheless, their beneficial effects predominantly outweigh the risks, sustaining ongoing research into phenolic compounds for cancer therapy. Notably, phenolic acids demonstrate promising therapeutic potential for MASLD, with CiA exhibiting no hepatotoxicity, embryotoxicity, or metabolic accumulation risks, suggesting suitability for pregnant populations. CGA, while possessing the lowest bioavailability, offers the widest safety margin; its limited systemic absorption paradoxically reduces toxicity, and dosage form optimization may overcome efficacy limitations. Documentation of adverse effects, contraindications, and off-target effects across individual phenolic acids should be prioritized in subsequent clinical research to establish evidence-based safety frameworks for complex metabolic disease management.

## 5. The Current Found and Limitations of Phenolic Acids in MASLD

**Figure 10:**
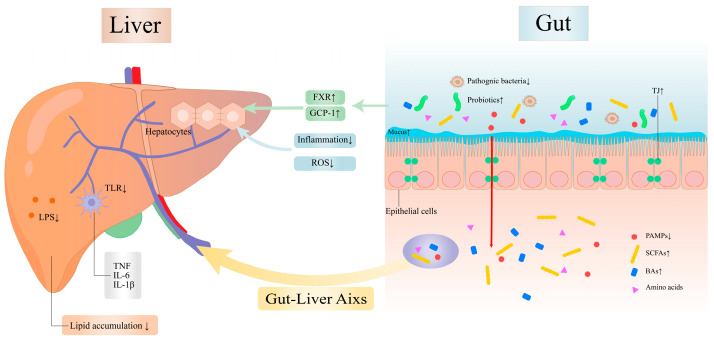
Mechanisms of the phenolic acids regarding MASLD. ↓ indicates a decrease in expression and ↑ indicates an increase in expression.

The aforementioned phenolic acids demonstrate therapeutic potential for MASLD (Figure 10), with comparative analysis revealing that all modulate MASLD through regulation of antioxidant activity and NF-κB inflammatory pathway. Their antioxidant capacity depends on the number and position of hydroxyl substituents on the benzene ring: structures with ortho-dihydroxy groups or 4-hydroxy-3-methoxy moieties exhibit enhanced activity radical stabilization and metal chelation capabilities, whereas unsubstituted CiA shows weak antioxidant effects [98]. The NF-κB pathway represents a common target, with multiple phenolic acids inhibiting this signaling cascade via the canonical pathway, thereby downregulating NF-κB and its mediated pro-inflammatory cytokines, as observed for CiA, PCA, and GA. CGA, CaA, and FA suppress inflammation through MAPK pathway inhibition. VA inhibits NLRP3 inflammasome activation by downregulating caspase-1 and NLRP3 expression [218]. Select phenolic acids further exert anti-inflammatory effects by promoting Nrf2 activation.

Each phenolic acid possesses distinct biological activities. MCT selectively mediates the active absorption of pCA and FA, while CiA interacts with hepatic transporters such as MDR1 and NTCP. Inhibition of these transporters reduces its plasma concentration. Nuclear receptors serve as key regulatory targets for select phenolic acids: CGA enhances FXR expression to modulate bile acid metabolism. FA upregulates PPARα to promote fatty acid oxidation. GA activates the AMPK-PPARα axis to reduce lipid accumulation. Furthermore, the gut–liver axis triggers local and distal responses countering disease pathogenesis. Phenolic acids ameliorate MASLD through multi-target modulation of the gut–liver axis. CiA, FA, and VA upregulate SCFA-producing bacteria while downregulating LPS-producing bacteria. FA and CGA promote intestinal villi development and upregulate tight junction proteins (occludin, ZO-1) to reinforce gut barrier integrity. VA inhibits ferroptosis via CA9 binding, preventing excessive enterocyte death. CGA improves FXR-mediated bile acid homeostasis, optimizing enterohepatic circulation. Concurrently, phenolic acids synergistically suppress gut–liver inflammation. SCFAs act as pivotal anti-inflammatory mediators that activate PRRs to mitigate hepatic inflammation and lipid accumulation.

Despite the multi-pathway therapeutic potential of phenolic acids in MASLD, current research exhibits significant contradictions and limitations. Comparative analysis reveals that pCA mechanisms in MASLD predominantly derive from combination therapy studies, obscuring its precise mechanistic contributions. Research on CA and VA remains scarce, lacking robust evidence to elucidate their modes of action. Phenolic acids exhibit inhibitory effects on certain cytochrome P450 enzymes [219]. However, conflicting reports exist regarding FA’s modulation of CYP7A1, with two studies yielding diametrically opposed outcomes. Model relevance concerns persist: evidence for CGA-mediated GLP-1 secretion originates from rotenone-induced Parkinson’s disease models, raising questions about MASLD applicability. FA iron metabolism data from iron-overloaded mouse models requires validation in MASLD contexts. Notably, rodent studies predominantly utilize male subjects, a design choice that prioritizes hormonal stability and data consistency. This aligns with the higher prevalence of MASLD in human males [220]. The substantial interindividual diversity of human gut microbiota poses reproducibility challenges for phenolic acid-induced alterations in microbial abundance during clinical trials. Concurrently, in vivo experiments need to address species-specific differences, biliary excretion pathways may significantly diverge between rat models and humans, and some excretion data derived from rats limits clinical extrapolation. Current research predominantly relies on preclinical models (cellular/animal), with clinical studies remaining scarce and evidence insufficient, creating a translational evidence gap. These limitations underscore the necessity for rigorous clinical validation and real-world evidence alongside mechanistic exploration.

Current research exhibits significant gaps in exploring synergistic effects between phenolic acids and standard MASLD therapies, such as PPAR agonists or lifestyle interventions. For instance, mechanistic synergies remain unvalidated. Phenolic acids alleviate inflammation via NF-κB pathway inhibition, while PPAR agonists improve metabolism through lipid oxidation activation. Their potential “anti-inflammatory-metabolic regulation” dual-pathway synergy lacks experimental assessment in combined regimens. Studies on polyphenol combinations are absent. However, different phenolic acids may target complementary pathways, suggesting potential for compound formulations addressing MASLD’s multifaceted pathology. In addition, clinical trials evaluating phenolic acids combined with clinically approved drugs should be initiated. Through such strategies, synergistic value in MASLD comprehensive management can be systematically explored, overcoming current monotherapy limitations in addressing disease heterogeneity.

## 6. Conclusions

The pathophysiology of MASLD is complex and involves interactions and dependencies between the liver and other organs (particularly the kidneys, intestines, and adipose tissues). Therefore, the development of effective treatments has become a prominent research focus. Extensive studies have revealed that phenolic acid compounds exhibit significant potential in ameliorating MASLD through multiple mechanisms. This review comprehensively reviews the latest research advances on HCAs and HBAs in MASLD intervention. These phenolic acids have powerful antioxidant and anti-inflammatory properties, and protect the liver directly or indirectly through the gut–liver axis. Although phenolic acids have demonstrated efficacy in ameliorating MASLD, there are still a lot of significant challenges remaining to be addressed before their clinical translation as therapeutic agents. Phenolic acids represent a promising therapeutic avenue for MASLD management, warranting further in-depth exploration to fully characterize their pharmacological profiles, therapeutic efficacy, and safety parameters.

## Figures and Tables

**Table 1:** Clinical trials and drugs in the treatment of MASLD.

Agent (Trial Name)	Primary Mechanism	Most Frequent Adverse Events	Trial Number	Ref.
Silymarin	Antioxidant	No obvious adverse events	NCT02006498	[30]
Fucoxanthin	Bioactive dietary supplement	No obvious adverse events	NCT02875392	[31]
Aspirin	Cox inhibitor	Upper respiratory infection, gastrointestinal disorders	NCT04031729	
Obeticholic Acid	FXR agonist	Pruritus, fatigue, gastrointestinal disorders	NCT02633956	[32]
EDP-305	FXR agonist	Pruritus, fatigue	NCT03421431	[33]
EYP001a	FXR agonist	Pruritus, fatigue	NCT03812029	[34]
Pitavastatin	HMG-CoA reductase inhibitor	The risk of diabetes, gastrointestinal disorders	NCT02290106	[35]
Vitamin E	Lipid-soluble antioxidant	Iron deficiency or anemia	NCT01792115	[36]
Saroglitazar Magnesium	PPAR α/δ Agonist	Pruritus	NCT03863574	[37]
Metformin	Suppresses HGP	Weight loss	NCT00063232	[38]

HGP: hepatic glucose production.

**Table 2:** Mechanistic role of HCAs in MASLD.

HCAs	Structure	Effects	Model	Dose	Mechanisms of Action	Ref.
Cinnamic acid (C_9_H_8_O_2_)	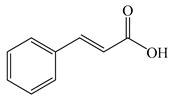	Anti-inflammatory	HFD-induced hyperlipidemia in rats	30 mg/kg PO for 49 days	↓ Serum leptin, pancreatic lipase	[51]
			HFFD-induced MASLD in rats	10, 20, 40 mg/kg PO for 70 days	↓ TNF-α	[52]
		Hepatoprotection	HFD-induced hyperlipidemia in rats	30 mg/kg PO for 49 days	↓ TC, TG, LDL-C	[51]
			OA-induced HepG2	12.5, 25, 50, 100, 200 μM for 24 h	↓ TG, FFA, IHTG	[53]
			db/db mice	20 mg/kg PO for 28 days		
			db/db mice	20 mg/kg PO for 28 days	↑ ATP1B1, LDLR, 5-HT ↓ TG, FFA	[54]
			HFD-induced MASLD in rats	23 g/kg PO for 56 days	↑ MDR1, NTCP, OATP1A2, OCT1, MATE1 ↓ TC, HDL-C	[55]
		Intestinal barrier integrity	Loperamide-induced STC in mice	40, 80 mg/kg PO for 28 days	↑ *Firmicutes*, SCFAs, goblet cells	[56]
p-coumaric acid (C_9_H_8_O_3_)	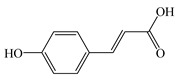	Antioxidant	APAP-induced hepatotoxicity in mice	50 mg/kg PO for 7 days	↓ ROS	[57]
		Anti-inflammatory	APAP-induced hepatotoxicity in mice	50 mg/kg PO for 7 days	↓ MAPK, NF-κB, TNF-α, IL-1β, IL-6	[57]
			Dust + LPT rats and Dust + IR rats	100 mg/kg IP for 42 days	↓ NF-κB, TNF-α, IL-1β, IL-6	[58]
			HFD-induced MASLD in rats	200 mg/kg PO for 70 days	↓ NF-κB, TNF-α	[59]
			HFHS-induced MASLD in mice	50 mg/kg PO for 84 days	↓ TNF-α, IL-6, IL-8, insulin, adiponectin, serum leptin	[60]
		Hepatoprotection	Dust + LPT rats and Dust + IR rats	100 mg/kg IP for 42 days	↓ TC, TG, LDL-C, HDL-C	[58]
			HFD-induced MASLD in rats	200 mg/kg PO for 70 days	↓ TG	[59]
			Triton WR1339-induced hyperlipidemia HepG2	10 μg/mL	↑ AMPK, PPARα/γ ↓ TC, TG	[61]
			Triton WR1339-induced hyperlipidemia in mice	100 mg/kg PO		
Caffeic acid (C_9_H_8_O_4_)	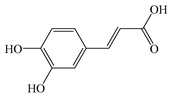	Anti-inflammatory	DSS-induced colitis in mice	179 mg/kg PO 14 days	↑ IL-4, CYP4B1 ↓ L-17, iNOS	[62]
			DSS-induced colitis in mice	1 mM PO for 23 days	↓ NF-κB, IL-6, TNF-α, IFNγ	[63]
		Hepatoprotection	Alloxan-induced type 1 diabetic in mice	50 mg IP for 7 days	↓ TC, TG, LDL-C, HDL-C	[64]
Ferulic acid (C_10_H_10_O_4_)	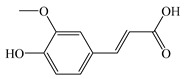	Antioxidant	APAP-induced inflammation in mice	10, 30, 100 mg/kg PO 3 times	↓ SOD, CAT	[65]
			HCHF-induced metabolic syndrome in rats	30, 60 mg/kg PO 42 days	↑ HO-1, HSP70, Akt ↓ COX-2	[66]
			Iron-induced mice	50 mg/kg PO 16 days	↓ ROS	[67]
		Anti-inflammatory	APAP-induced inflammation in mice	10, 30, 100 mg/kg PO 3 times	↓ p38MAPK, NF-κB, TLR4, IL-1β	[65]
			CCI-induced mice	100 mg/kg PO 21 days	↓ TLR4, NF-κB, IL-1β, IL-6	[68]
			HFD-induced mice	100 mg/kg PO 84 days	↓ NF-κB, TLR4	[69]
		Hepatoprotection	APAP-induced inflammation in mice	10, 30, 100 mg/kg PO 3 times	↓ CYP2E1	[65]
			HFD-induced mice	100 mg/kg PO 84 days	↑ SCFAs ↓ LPS	[69]
			HFD-induced mice	100 mg/kg PO 84 days	↑ CYP7A1, BAs ↓ TC	[70]
			Iron-induced mice	50 mg/kg PO 16 days	↓ FASN, CYP7A1, TC, BAs	[67]
			HFD-induced mice	100 mg/kg PO 84 days	↑ PPARα	[71]
		Intestinal barrier integrity	HS-induced IEC-6 cells	1, 5, 10, 20, 50, 100, 200, 500 μM for 48 h	↑ ZO-1, occludi, E-cadherin	[72]
			HS-induced rats	50 mg/kg PO 7 days		
Chlorogenic acid (C_16_H_18_O_9_)	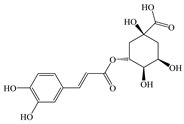	Antioxidant	MCDD-induced MASLD in mice	30, 60 mg/kg PO 28 days	↑ Nrf2, PPARα	[73]
			Weaned piglets	1000 mg/kg	↑ Nrf2, HO-1	[74]
		Anti-inflammatory	HFD-induced colitis in mice	100 mg/kg PO 105 days	↓ NF-κB, TLR4	[75]
			LPS-induced inflammation in mice	12.5, 25, 50 mg/kg IP	↓ NF-κB, TNF-α, IL-1β, IL-6	[76]
			Indomethacin-induced mice	50 mg/kg PO 12 days	↓ *Bacteroides*, LPS	[77]
			MCDD-induced MASLD in mice	30, 60 mg/kg PO 28 days	↓ TLR4	[73]
			Weaned piglets	1000 mg/kg	↓ NF-κB, TLR4, IL-1β, IL-6	[74]
			Chronic stress-induced rats	100 mg/kg PO	↓ p38MAPK, NF-κB	[78]
			HUA-induced mice	30, 60 mg/kg PO 19 days	↓ IL-1β, IL-6	[79]
		Hepatoprotection	HFS-induced mice	50, 100, 200 mg/kg PO	↑ FXR, BAs	[80]
			HFD-induced MASLD in mice	1.34 mg/kg PO 28 days	↑ FXR, SHP, BSEP, FGF15	[81]
			HFD-induced colitis in mice	100 mg/kg PO 105 days	↑ PPAR-α, BCAAs, SCFAs ↓ LPS	[75]
			MCDD-induced MASLD in mice	30, 60 mg/kg PO 28 days	↑ PPARα ↓ LPS	[73]
		Intestinal barrier integrity	HUA-induced mice	30, 60 mg/kg PO 19 days	↑ ZO-1, occludi, SCFAs	[79]

↓ indicates a decrease in expression and ↑ indicates an increase in expression. APAP: acetaminophen/N-acetyl-p-aminophenol; BAs: bile acids; BCAAs: branched-chain amino acids; BSEP: bile salt export pump; CAT: catalase; CCI: constriction injury; COX-2: cyclooxygenase-2; CYP7A1: cholesterol 7-α hydroxylase; DSS: dextran sulfate sodium; FGF: fibroblast growth factor; HCHF: high-carbohydrate, high-fat diet group; HFD: high-fat diet; HFFD: high-fat, high-fructose diet; HFS: 20% fructose in drinking water plus 4% sodium chloride in the diet; HO-1: heme oxygenase-1; HS: heat stress; HSP70: heat shock protein 70; HUA: hyperuricemia; MCDD: methionine-choline-deficient diet; SCFAs: short-chain fatty acids; SHP: small heterodimer partner; TLR4: toll-like receptor 4; ZO-1: zonula occludens-1.

**Table 3:** Mechanistic role of HBAs in MASLD.

HBAs	Structure	Effects	Model	Dose	Mechanisms of Action	Ref.
Protocatechuic acid (C_7_H_6_O_4_)	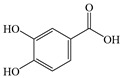	Antioxidant	Menadione-induced hepatotoxicity in rats	10, 20 mg/kg PO 7 days	↑ CAT, SOD, Nrf2	[150]
			HepG2	0, 25, 50 μM for 24 h	↓ ROS, caspase-3/7, PARP, ER stress	[151]
			HFD-induced MASLD in mice	30 mg/kg PO 56 days	↓ ROS	[152]
			HFD-induced MASLD in rats	10, 20 mg/kg PO 56 days		
		Anti-inflammatory	Synovial membrane tissues	5, 10, 20, 40 μM for 24, 48 h	↓ NF-κB, TNF-α, IL-1β, IL-6	[153]
		Hepatoprotection	HFD-induced MASLD in mice	30 mg/kg PO 56 days	↑ SIRT3 ↓ ACSF3	[152]
			HFD-induced MASLD in rats	10, 20 mg/kg PO 56 days		
			HFD-induced MASLD in mice	0.25, 0.5, 1 g/kg RRBE PO 84 days	↓ TC, FFA, LDL-C, LXRα, ATGL, CD36	[154]
			HFD-induced MASLD in mice	HFD containing 0.4% of PCA for 84 days	↑ FGF1, CPT1α ↓ *Enterococcus faecalis*	[155]
			HFD-induced MASLD in mice	HFD containing 200 mg/kg/20 mL of PCA for 42 days	↑ HDL, total thiol ↓ TC, TBARS, liver enzymes	[156]
Vanillic acid (C_8_H_8_O_4_)	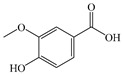	Antioxidant	H_2_O_2_-induced D.Mel-2 cell	125, 250 μg/mL 24 h	↑ CAT, SOD ↓ ROS	[157]
		Anti-inflammatory	IL-1β, TNF-α-induced chondrocytes	1 μM 72 h	↓ NF-κB, IL-1β, TNF-α	[158]
		Hepatoprotection	FL83B	20 μL 6.25 ng/mL	↑ PI3K, GLUT-2, phosphorylated ACC	[159]
			HFD-induced mice	30 mg/kg PO 28 days		
			HFD-induced MASLD in rats	50 mg/kg PO 56 days	↑ FGF21 ↓ TC, TG, P62	[160]
			CCl4 and olive oil-induced liver fibrosis in rats	5, 20 mg/kg PO 56 days	↓ MIF, CD74, LC3B	[161]
		Intestinal barrier integrity	Weaned piglets	4000 mg/kg PO 21 days	↑ Oclaudin, *Firmicutes/Bacteroidetes* ratio ↓ LPS	[162]
			DSS-induced colitis in mice	12.5, 25, 50 mg/kg PO 7 days	↑ SREBP1, SCD1	[163]
Gallic acid (C_7_H_6_O_5_)	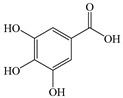	Anti-inflammatory	IL-1β-induced HIEC-6	20, 40, 60 mg/mL 48 h	↑ IL-4, IL-10	[164]
			TNBS-induced colitis in mice	20, 40, 60 mg/kg PO 7 days	↓ TGF-β, TNF-α, IL-1, IL-6, IL-12	
		Hepatoprotection	PA-induced HepG2, Hepa 1-6, RAW 264	50, 200 μM 24 h	↑ AMPK ↓ SREBP-1c, LXRα, CD36, FATP2, ACCα, caspase 3/7	[165]
			OA/PA-induced HepG2, SMMC-7721	20 μM 24 h	↑ AMPK, PPARα	[166]
			HepG2	0, 10, 20, 40 μM 24 h	↑ EGFR-ERK1/2, LDLR	[167]

↓ indicates a decrease in expression and ↑ indicates an increase in expression. ATGL: adipose triglyceride lipase; CAT: catalase; CD36: cluster of differentiation 36; CPT1α: carnitine palmitoyltransferase-1 alpha; GLUT-2: glucose transporter-2; LC3B: microtubule-associated protein 2 light chain 3 type B; MIF: macrophage migration inhibitory factor; PA: palmitic acid; PARP: poly (ADP-ribose) polymerase; PI3K: phosphoinositide 3-kinase; RRBE: red rice bran extract; SOD: superoxide dismutase; TGF-β: transforming growth factor-beta.

## Abbreviations

The following abbreviations are used in this manuscript:

ACC	Acetyl-CoA carboxylase
ACSF3	Acyl-CoA synthetase family member 3
Akt	Protein kinase B
AMPK	AMP-activated protein kinase
APAP	Acetaminophen/N-acetyl-p-aminophenol
ATGL	Adipose triglyceride lipase
BAs	Bile acids
BCAAs	Branched-chain amino acids
BSEP	Bile salt export pump
CaA	Caffeic acid
CAPE	Caffeic acid phenethyl ester
CAT	Catalase
CA9	Carbonic anhydrase IX
CCI	Constriction injury
CD36	Cluster of differentiation 36
CGA	Chlorogenic acid
CiA	Cinnamic acid
COX-2	Cyclooxygenase-2
CPT1α	Carnitine palmitoyltransferase-1 alpha
CYP7A1	Cholesterol 7-α hydroxylase
DSS	Dextran sulfate sodium
ECM	Extracellular matrix
ED_50_	Median effective dose
EGFR	Epidermal growth factor receptor
ER	Endoplasmic reticulum
ERK	Extracellular regulated protein kinase
EVs	Extracellular vesicles
FA	Ferulic acid
FASN	Fatty acid synthase
FATP2	Fatty acid transport protein 2
FFA	Free fatty acid
FGF	Fibroblast growth factor
FXR	Farnesoid X receptor
GA	Gallic acid
GLP-1	Glucagon-like peptide-1
GLUT-2	Glucose transporter-2
HBAs	Hydroxybenzoic acids
HCAs	Hydroxycinnamic acids
HCC	Hepatocellular carcinoma
HCHF	High-carbohydrate, high-fat diet group
HCs	Hepatocytes
HDL	High-density lipoprotein
HFD	High fat diet
HFFD	High-fat, high-fructose diet
HFS	20% fructose in drinking water plus 4% sodium chloride in the diet
HGP	Hepatic glucose production
HO-1	Heme oxygenase-1
HS	Heat stress
HSCs	Hepatic stellate cells
HSP70	Heat shock protein 70
HUA	Hyperuricemia
IGFBP-2	Insulin-like growth factor binding protein-2
IHTGs	Intrahepatic triglycerides
IL-1β	Interleukin-1β
IL-6	Interleukin-6
INSIG2	Insulin-induced gene 2
IR	Insulin resistance
IRS1	Insulin receptor substrate 1
IRS2	Insulin receptor substrate 2
KCs	Kupffer cells
LC3B	Microtubule-associated protein 2 light chain 3 type B
LDL	Low-density lipoprotein
LDL-C	Low-density lipoprotein cholesterol
LDLR	Low-density lipoprotein receptor
LD_50_	Median lethal dose
LPS	Lipopolysaccharides
LSECs	Liver sinusoidal endothelial cells
LTA	Lipoteichoic acid
LXRα	Liver X receptor alpha
MAPK	Mitogen-activated protein kinase
MAFLD	Metabolic Dysfunction-Associated Fatty Liver Disease
MASLD	Metabolic Dysfunction-Associated Steatotic Liver Disease
MCDD	Methionine-choline-deficient diet
MCT	Monocarboxylate transporter
MIF	Macrophage migration inhibitory factor
MLCK	Myosin light chain kinase
MyD88	Myeloid differentiation primary response 88
NAFL	Non-alcoholic fatty liver
NAFLD	Non-alcoholic fatty liver disease
NASH	Non-alcoholic steatohepatitis
NF-κB	Nuclear factor κB
Nrf2	Nuclear factor-erythroid 2-related factor 2
PA	Palmitic acid
PAMPs	Pathogen-Associated Molecular Patterns
PARP	Poly(ADP-ribose) polymerase
pCA	p-coumaric acid
PCA	Protocatechuic acid
PI3K	Phosphoinositide 3-kinase
PPARs	Peroxisome proliferator-activated receptors
ROCK1	Rho-associated kinase 1
ROS	Reactive oxygen species
RRBE	Red rice bran extract
PRRs	Pattern Recognition Receptors
SCAP	SREBP cleavage-activating protein
SCD1	Stearoyl-CoA desaturase-1
SCFAs	Short-chain fatty acids
SHP	Small heterodimer partner
SIRT3	Sirtuin 3
SOD	Superoxide dismutase
SREBP-1c	Sterol regulatory element-binding protein-1c
TBARS	Thiobarbituric acid reactive substances
TC	Total cholesterol
TCA	Trans-cinnamic acid
TG	Triglyceride
TGF-β	Transforming Growth Factor-beta
TI	Therapeutic index
TJ	Tight junctions
TLRs	Toll-like receptors
TLR4	Toll-like receptor 4
TNF-α	Tumor necrosis factor-α
TZDs	Thiazolidinediones
T2DM	Type 2 diabetes mellitus
VA	Vanillic acid
ZO-1	Zonula occludens-1
5-HT	5-hydroxytryptamine
